# Eutectogel Electrolyte Constructs Robust Interfaces for High‐Voltage Safe Lithium Metal Battery

**DOI:** 10.1002/advs.202310136

**Published:** 2024-04-19

**Authors:** Wanbao Wu, Deping Li, Chaochao Gao, Hao Wu, Yiyang Bo, Jichuan Zhang, Lijie Ci, Jiaheng Zhang

**Affiliations:** ^1^ Sauvage Laboratory for Smart Materials Harbin Institute of Technology Shenzhen 518055 China; ^2^ School of Petrochemical Engineering Changzhou University Changzhou 21300 China; ^3^ Changzhou Qianmu New Energy Co. Ltd. Changzhou 21300 China; ^4^ School of Materials Science and Engineering Harbin Institute of Technology Shenzhen 518055 China

**Keywords:** eutectogel electrolyte, high voltage, LCO cathode, lithium metal batteries, structure/interface stability

## Abstract

Dramatic growth of lithium dendrite, structural deterioration of LiCoO_2_ (LCO) cathode at high voltages, and unstable electrode/electrolyte interfaces pose significant obstacles to the practical application of high‐energy‐density LCO||Li batteries. In this work, a novel eutectogel electrolyte is developed by confining the nonflammable eutectic electrolyte in a polymer matrix. The eutectogel electrolyte can construct a robust solid electrolyte interphase (SEI) with inorganic‐rich LiF and Li_3_N, contributing to a uniform Li deposition. Besides, the severe interface side reactions between LCO cathode and electrolyte can be retarded with an in situ formed protective layer. Correspondingly, Li||Li symmetrical cells achieve highly reversible Li plating/stripping over 1000 h. The LCO||Li full cell can maintain 72.5% capacity after 1500 cycles with a decay rate of only 0.018% per cycle at a high charging voltage of 4.45 V. Moreover, the well‐designed eutectogel electrolyte can even enable the stable operation of LCO at an extremely high cutoff voltage of 4.6 V. This work introduces a promising avenue for the advancement of eutectogel electrolytes, the nonflammable nature and well‐regulated interphase significantly push forward the future application of lithium metal batteries and high‐voltage utilization of LCO cathode.

## Graphical abstract

A well‐designed eutectogel electrolyte constructs a robust solid electrolyte interphase with inorganic‐rich LiF and Li_3_N, contributing to a uniform Li deposition. The severe interface side reactions between LiCoO_2_ cathode and electrolyte can be retarded with an in situ formed protective layer. The well‐regulated interphases ensure the long cycling performance of high energy density LCO||Li cells at high voltage.

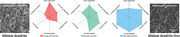

## 1 Introduction

With the rapid advancements in intelligence and information technology, the pursuit of smart, lightweight design, and extended cycle life emerges as a primary focus in the field of portable electronics (e.g., mobile phones, laptops, and digital cameras).^[^ ^1^, ^2^, ^3^ ^]^ These characteristics demand higher volumetric energy densities for lithium‐ion batteries. The most promising approaches to achieve this goal are to increase the cathode/anode capacities or to increase the operating voltage.^[^ ^4^, ^5^, ^6^ ^]^ This is due to the material compacted density, the mass ratio of active material, the thickness of the separators, and the current collectors are already close to their limits and the energy density that can be increased is very limited. In addition, reducing the thickness of the separators and the current collector would increase the safety risks.^[^ ^7^ ^]^

From the perspective of cathode materials, enhancing the energy density of Li‐ion batteries presents an ongoing challenge. LiCoO_2_ (LCO) cathode still has a definite advantage owing to its easy synthesis, high compact density, high initial Coulomb efficiency, high operating voltage, and good cycling stability. Although the theoretical capacity of the LCO cathode is as high as 275 mAh g^−1^, the practical capacity is only 140 mAh g^−1^ at a cutoff voltage of 4.2 V. By increasing the cutoff voltage to 4.5 V, the capacity can be increased to 180 mAh g^−1^.^[^ ^8^ ^]^ However, the practical application of high‐voltage LCO still faces huge challenges, suffering from structural collapse and detrimental cathode/electrolyte interface reactions when the cutoff voltage exceeds 4.5 V, leading to rapid decay.^[^ ^9^, ^10^, ^11^ ^]^ Therefore, the establishment of a highly stable cathode–electrolyte interphase (CEI) is essential to improve the performance of high‐voltage LCO cathodes. Many substantial efforts have been devoted to stabilizing the surface structure of LCO cathode and cathode/electrolyte interface at high voltage, such as cation doping,^[^ ^12^, ^13^, ^14^ ^]^ surface modification,^[^ ^15^, ^16^, ^17^ ^]^ separator improvement^[^ ^18^, ^19^, ^20^ ^]^ and electrolyte modification.^[^ ^21^, ^22^, ^23^ ^]^ Among these, the in situ formation of stable LiCoO_2_/electrolyte interface films by electrolyte design stands out as a promising and cost‐effective approach, although it requires further refinement. Conventional carbonate and ether electrolytes are prone to decomposition under high voltage, resulting in inferior cycling performance.^[^ ^24^, ^25^ ^]^ In particular, the high flammability of carbonate electrolytes is still a big challenge. Eutectic electrolyte delivers high safety with high thermal stability and nonflammability. In addition, polymer gel electrolytes have received increasing attention due to good interfacial contact with lithium metal anode, high conductivity, and effective inhibition of lithium dendrite growth. Combining polymer matrix with eutectic electrolyte provides a new strategy for realizing high safety and high energy density lithium metal batteries.^[^ ^26^, ^27^, ^28^ ^]^ Consequently, the development of a new eutectogel electrolyte to match high‐voltage LCO cathodes and deliver higher capacity is imperative.

Herein, we designed a succinonitrile (SN)‐based eutectic electrolyte containing lithium bis(trifluoromethanesulfonyl)imide (LiTFSI), lithium borate difluorooxalate (LiDFOB), and SN in eutectic ratio and fluoroethylene carbonate as an additive. Due to its high dielectric constant, SN exhibits superior capabilities to dissolve lithium salts and form eutectic solutions.^[^ ^29^, ^30^ ^]^ Notably, we have also developed a eutectogel electrolyte by encapsulating the eutectic electrolyte within a polymer matrix, which enhances the interfacial stability with the Li metal anode and enables long‐term stable Li stripping and plating. A synergistic stabilization of the lithium metal anodes and LCO cathodes is achieved by a stable interface formed with the eutectogel electrolyte, which prevents excessive electrolyte decomposition and ensures structural stability of the LCO cathode at high voltages. Moreover, comprehensive scanning electron microscopy, transmission electron microscopy, and X‐ray photoelectron spectroscopy characterizations were introduced to further decipher the microstructure of the CEI. This work provides valuable insights into the feasibility of eutectogel electrolytes to achieve high safety and high energy density LCO||Li batteries.

## 2 Results and Discussion

**Figure 1:**
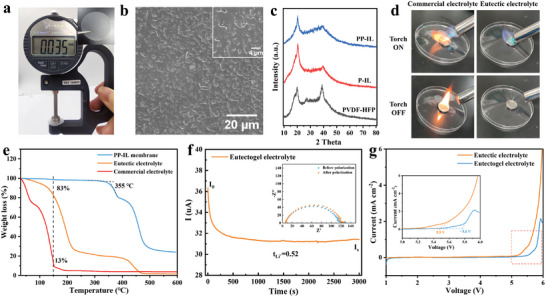
a) Optical photograph and thickness of eutectogel electrolyte. b) SEM images of eutectogel electrolyte. c) XRD patterns of PVDF‐HFP, P‐IL, and PP‐IL membranes. d) Flammability testing of eutectic electrolyte and commercial electrolyte. e) TGA of commercial electrolyte, eutectic electrolyte, and PP‐IL membrane. f) Current–time curve of Li/eutectogel electrolyte/Li symmetrical cells with a 5 mV DC voltage applied. g) LSV plots of eutectic electrolyte and eutectogel electrolyte.

The preparation of the eutectogel electrolyte is visually illustrated in Figure S1 (Supporting Information) and consists of two main steps. The first stage involves immobilizing 1‐ethy‐3‐methylimidazolium bis(trifluoromethylsulfonyl)imide (EMITFSI) within a copolymer gel framework. This is accomplished by casting a mixed acetone solution containing EMITFSI, poly(vinylidene fluoride‐co‐hexafluoropropylene) (PVDF‐HFP), and polyethylene oxide (PEO) to produce a thin and uniform film, ≈35 µm thick, with a macroporous structure measuring hundreds of nanometers on the surface (**Figure** **1**a,b), termed as PP‐IL. For comparison, PVDF‐HFP membranes without EMITFSI and PEO (termed as PVDF‐HFP membranes), and PVDF‐HFP membranes with EMITFSI but without PEO (termed as P‐IL) were also prepared. Subsequently, the PP‐IL film was immersed in a eutectic electrolyte consisting of LiTFSI:LiDFOB:SN with a molar ratio of 0.8:0.2:10 and 10 wt.% FEC was added as a film‐forming additive to form a eutectogel electrolyte. Notably, the eutectic electrolyte has a high ionic conductivity of 3.62 mS cm^−1^. The average eutectic electrolyte adsorption of the PP‐IL membrane is 11.2% (Table S1, Supporting Information). The incorporation of EMITFSI and PEO induces a reduction in the crystallinity and an augmentation of the amorphous phase within PVDF‐HFP,^[^ ^31^ ^]^ as depicted in Figure 1c. Notably, the introduction of EMITFSI enhances the stretching capacity of the polymer membrane from 66.7% to 80.0%, rendering the membrane considerably more flexible (Figure S2, Supporting Information).

For PP‐IL membranes, the strong ionic dipole interactions between imidazole cations and PVDF‐HFP and PEO give them a certain self‐healing ability.^[^ ^32^ ^]^ As represented in Figure S3 (Supporting Information), the PP‐IL film was scratched with a razor blade and immersed in the eutectic electrolyte. After soaking for 12 h, the scratch on the PP‐IL membrane almost completely disappeared. Moreover, the peak shifts from IR spectra (Figure S4, Supporting Information) and the short distance of the atoms from DFT structure optimization (Figure S5, Supporting Information) confirm the strong ionic dipolar interaction between EMITFSI and PVDF and PEO.^[^ ^33^ ^]^ Therefore, EMITFSI not only reduces the crystallinity of the polymer but also cross‐links with the polymer matrix through a strong ionic dipole interaction, facilitating Li^+^ mobility and increasing ionic conductivity (0.63 mS cm^−1^ at 25 °C). Notably, the ionic conductivity of eutectogel electrolytes is higher than that of most other gel electrolytes (Table S2, Supporting Information).

Great significance is attributed to the thermal stability of the electrolyte concerning battery safety, as it plays a pivotal role in averting potential hazards like short‐circuits or thermal runaway during battery operation. As shown in Figure 1e, the commercial electrolyte has a weight loss of 87% at 150 °C, compared to only 17% for the eutectic electrolyte. The weight loss of eutectic electrolytes is mainly attributed to the volatilization of FEC and very few SN. Furthermore, it is noteworthy that the PP‐IL membrane demonstrates impressive thermal resilience, tolerating temperatures of up to 350 °C. Most importantly, flammability tests revealed the nonflammable nature of eutectic electrolytes, primarily stemming from the intrinsic nonflammability of SN (Figure 1d). In stark contrast, commercial electrolytes containing carbonate solvents can easily be ignited, posing a significant safety hazard. Consequently, eutectogel electrolytes offer a commendable level of safety assurance. The eutectogel electrolyte also exhibits a high lithium‐ion transference number of 0.52 (Figure 1f), which can effectively alleviate electrode polarization and allows the battery to operate at elevated current densities. Notably, the eutectic electrolyte has a higher Li^+^ transference number of 0.72 (Figure S6, Supporting Information). This difference can be attributed to the introduction of a polymer matrix into the eutectic electrolyte, which serves as a physical barrier to the movement of Li^+^, thereby lowering the Li^+^ transference number of the eutectogel electrolyte. Additionally, the eutectogel electrolyte has a wide electrochemical window up to 5.5 V, greater than the 5.2 V of the eutectic electrolyte (Figure 1g). This indicates that eutectogel electrolytes can be applied to most high‐voltage cathode materials, even the 4.6 V LCO cathode.

**Figure 2:**
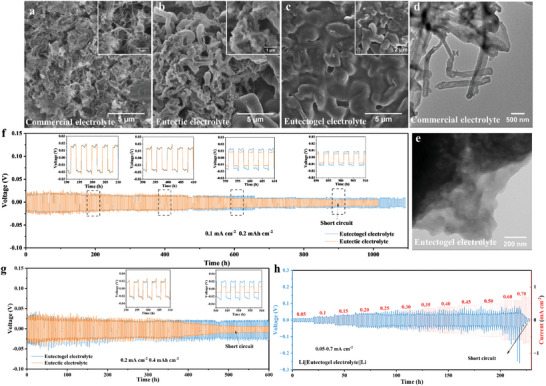
SEM images of Li metal deposited on Cu foil at 0.5 mA cm^−2^ with a capacity of 1.0 mAh cm^−2^ in a) commercial electrolyte, b) eutectic electrolyte, and c) eutectogel electrolyte. Cryo‐TEM images of lithium deposition in d) commercial electrolyte and e) eutectogel electrolyte. The cycling performance of Li–Li symmetrical cells with eutectic electrolyte and eutectogel electrolyte at f) 0.1 mA cm^−2^/0.2 mAh cm^−2^ and g) 0.2 mA cm^−2^/0.4 mAh cm^−2^. h) The voltage responses of Li||Li symmetric cells with eutectogel electrolyte under different current densities.

The morphology of deposited lithium was observed by scanning electron microscopy (SEM) for various electrolytes. As depicted in **Figure** **2**a–c, both the commercial electrolyte and eutectic electrolyte exhibit a porous structure with distinct lithium dendrites, but the eutectic electrolyte demonstrates an increased size. The presence of such microporous structures and lithium dendrites fosters ongoing side reactions between the active lithium metal and the electrolyte, leading to low Coulombic efficiency.^[^ ^34^ ^]^ Meanwhile, lithium dendrites can penetrate the separator during cell operation, posing a safety hazard. In sharp contrast, the eutectogel electrolyte shows a smooth and dense lithium deposition surface with large Li particles, which can ensure that detrimental side reactions between the lithium and the electrolyte are prevented (Figure 2c). The characterization of lithium deposition morphology was further substantiated by the use of cryo‐transmission electron microscopy (Cryo‐TEM), as depicted in Figure 2d,e. It is noteworthy that the commercial electrolyte displayed the presence of lithium dendrites with an approximate size of several hundred nanometers, signifying the potential risks associated with lithium metal batteries, such as short circuits and capacity fading. On the contrary, in the eutectogel electrolyte, a unique morphology was observed, consisting of a large number of stacked structures without any lithium dendrite, and this denser deposition morphology could reduce the side reactions between the electrolyte and lithium metal and stabilize the cell cycling. This observation not only confirms the SEM results but also suggests a more stable and safer lithium deposition behavior in eutectogel electrolytes.^[^ ^35^ ^]^

It is expected that dendrite‐free morphology will enhance the safety and lifespan of lithium metal batteries. The compatibility between eutectogel electrolyte and Li metal anode was further investigated by symmetric Li||Li cells under different current densities (Figure 2f,g). For the cells using eutectic electrolyte, a pronounced voltage drop occurs after 900 h at 0.1 mA cm^−2^ and 500 h at 0.2 mA cm^−2^, indicating an internal short circuit due to the continuous growth of Li dendrites (Figure 2b). As expected, the eutectogel electrolyte shows a stable voltage plateau over 1000 h at 0.1 mA cm^−2^ and 600 h at 0.2 mA cm^−2^. This enhanced cycling stability can be attributed to the dense stacked Li particle deposition, which ensures the reversibility of the lithium stripping/plating and effective suppression of side reactions (Figure 2c). Furthermore, the eutectogel electrolyte exhibits an ultimate current density of up to 0.6 mA cm^−2^ (Figure 2h).

The CE profile of Li plating/stripping was further conducted in Li||Cu cell. As shown in Figure S7 (Supporting Information), the carbonate electrolyte shows an average CE of only 74.02%, in contrast, eutectic electrolyte, and eutectogel electrolyte have higher average CE of 94.91% and 94.65%, respectively. In addition, the Coulombic efficiency of eutectogel electrolytes is higher than that of partial gel electrolytes, but still inferior to that of state‐of‐the‐art lithium metal electrolytes (Table S4, Supporting Information).

**Figure 3:**
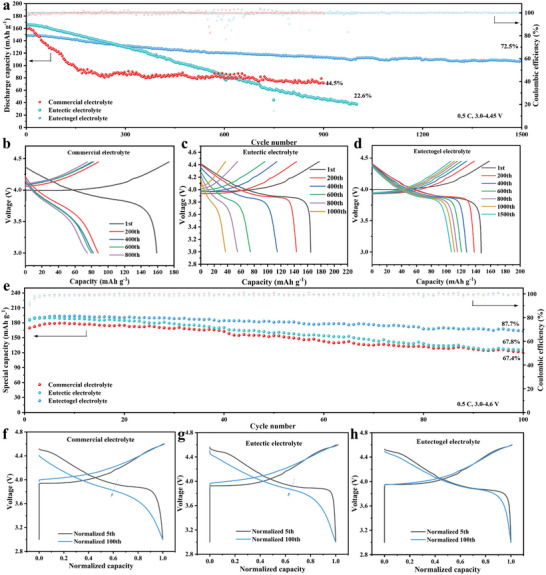
a) The cycling performance of LCO||Li cells utilizing various electrolytes under 3.0–4.45 V and 0.5C. The galvanostatic charge–discharge profiles of LCO||Li cells with different electrolytes at selected cycles in b) commercial electrolyte, c) eutectic electrolyte, and d) eutectogel electrolyte. e) The cycling performance of LCO||Li cells utilizing various electrolytes under 3.0–4.6 V and 0.5C. The normalized charge–discharge profiles at the fifth cycle and 100th cycle in f) commercial electrolye, g) eutectic electrolyte, and h) eutectogel electrolyte.

Benefits from the wide electrochemical window of the eutectogel electrolyte and good compatibility with lithium metal. Eutectogel electrolyte is applied to high voltage LCO||Li batteries. Figure S8 (Supporting Information) shows the rate performance of three electrolytes at room temperature from 3.0–4.45 V. The eutectogel electrolyte demonstrates slightly inferior rate performance compared to eutectic electrolytes and commercial electrolytes, especially at high rates. This performance discrepancy can be attributed to slower reaction kinetics resulting from its lower ionic conductivity. The long‐term cycling performance of LCO||Li cells was assessed at 0.5C and in the voltage range of 3.0–4.45 V (**Figure** **3**a). The eutectic electrolyte displays a high initial discharge capacity of 164.7 mAh g^−1^, but it decays rapidly, retaining 22.6% after 1000 cycles. Additionally, it is evident that the commercial electrolyte experiences rapid capacity fading after only 50 cycles, with a capacity retention of 41.8% after 1000 cycles. In contrast, the eutectogel electrolyte delivers superior long‐term cycling performance, boasting a capacity retention of 72.5%, an average CE as high as 99.9% after 1500 cycles, and a decay rate of only 0.018% per cycle. The charge–discharge profiles of eutectogel electrolytes also demonstrate less pronounced increases in voltage polarization after cycling compared to commercial electrolytes and eutectic electrolytes (Figure 3b,d). The reduced polarization voltage is due to the stable electrode/electrolyte interface. It is worth noting that, to the best of our knowledge, the long‐term cycling performance demonstrated in our work surpasses that of high‐voltage LCO cathodes within the 3.0–4.5 V range when using liquid or gel electrolytes (Table S5, Supporting Information).

To highlight the superiority of eutectogel electrolyte, we subjected the LCO||Li cells to cycling in 3.0–4.6 V (Figure 3e). In detail, the cell using eutectogel electrolyte displays the initial discharge capacity of 186.4 mAh g^−1^ at 0.5C with a remarkable capacity retention of 87.7% after 100 cycles, which is much better than in eutectic electrolyte (67.8%) and commercial electrolyte (31.8%). It is noteworthy that, as confirmed by the voltage spikes in Figure S9 (Supporting Information), an internal short circuit occurred after the 165th cycle in the case of the eutectic electrolyte (Figure S10, Supporting Information). This internal short circuit can be attributed to the continuous lithium dendrite growth. The normalized 5th and 100th charge/discharge curves reveal substantial voltage decay for the commercial electrolyte (Figure 3f) and eutectic electrolytes (Figure 3g), with the high voltage plateau from O3 to H1‐3 at 4.5 V disappearing after 100 cycles.^[^ ^4^ ^]^ In contrast, the eutectogel electrolyte exhibits minimal voltage decay after 100 cycles, and the high voltage plateau remains coincident with that observed during the fifth cycle (Figure 3h). In addition, the specific capacity from 4.5 to 4.6 V is calculated. The commercial electrolyte showed a decrease from 21 mAh g^−1^ in the fifth cycle to 14 mAh g^−1^ in the 50th cycle, while the eutectic electrolyte decreased from 27 mAh g^−1^ in the fifth cycle to 18 mAh g^−1^ in the 50th cycle. Remarkably, the eutectogel electrolyte remained stable after 50 cycles (Figure S11, Supporting Information). Furthermore, the potential for practical application of eutectogel electrolytes was verified by evaluating the performance of high‐loading LCO cathodes up to 10 mg cm^−2^. As shown in Figure S12 (Supporting Information), the capacity of the eutectogel electrolyte is 172.8 mAh g^−1^ after 15 cycles with a high‐loading LCO cathode between 3.0–4.45 V. The results demonstrate that eutectogel electrolytes can provide stable electrochemical performance even under high‐loading conditions.

**Figure 4:**
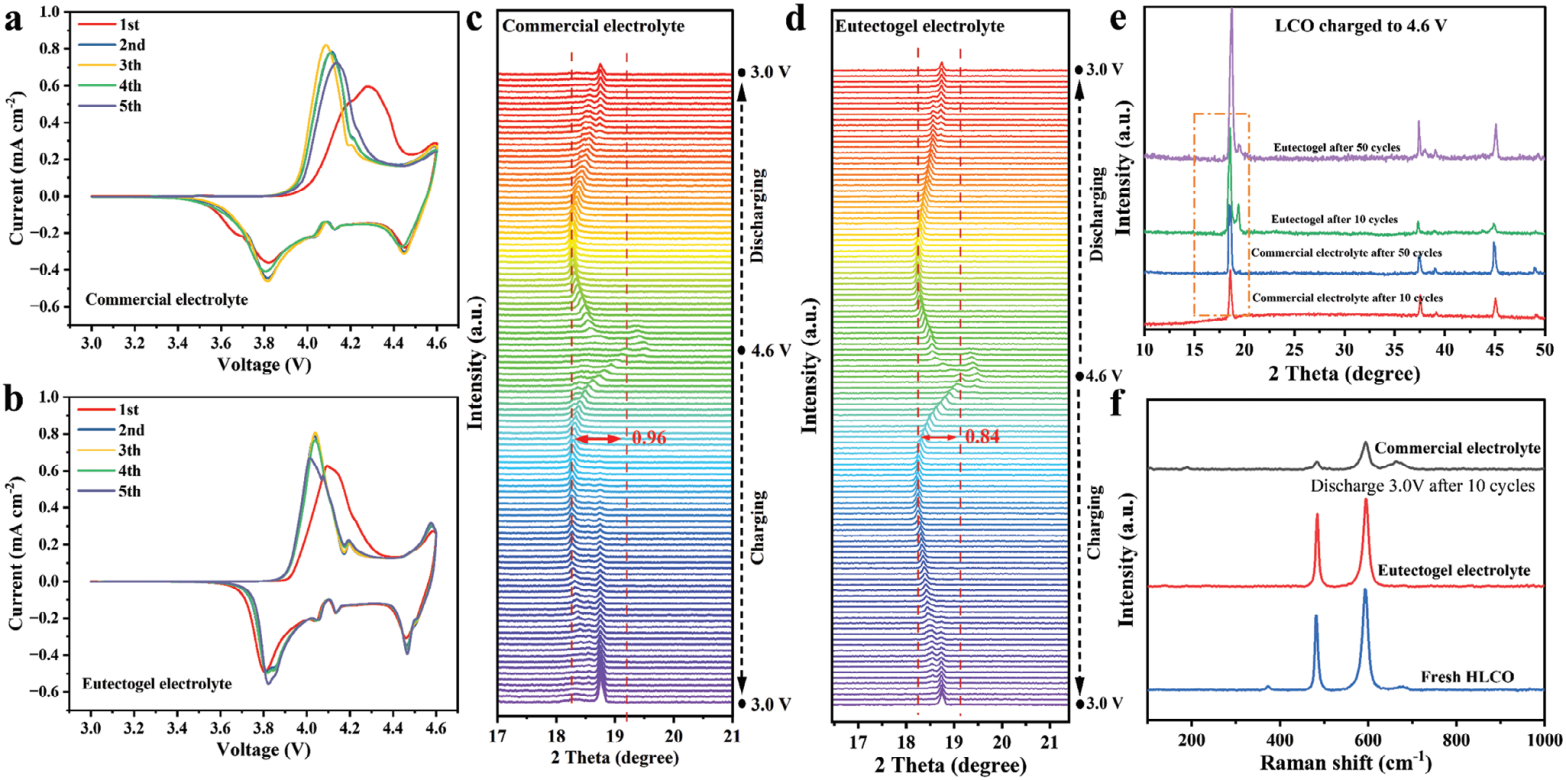
The Linear cyclic voltammetry curve for the first five cycles of LCO||Li cells between 3.0 and 4.6 V in a) commercial electrolyte and b) eutectogel electrolyte. In situ XRD curve of the first cycle of the LCO||Li between 3.0 and 4.6 V in c) commercial electrolyte and d) eutectogel electrolyte. e) XRD patterns of LCO cathode in commercial electrolytes and eutectogel electrolytes at different cycles. f) Raman spectra of the LCO cathode after ten cycles of different electrolytes.

The cell performance is strongly correlated with the structural evolution of the LCO cathode. **Figure** **4**a,b shows the CV profiles of the commercial electrolyte and the eutectogel electrolyte at 3.0–4.6 V. The oxide peak at 4.55 V corresponds to the phase transition from O3 to H1‐3, and the peak intensity gradually weakened in commercial electrolytes with cycling, indicating an irreversible phase transition in the LCO cathode.^[^ ^36^ ^]^ In sharp contrast, eutectogel electrolyte exhibits excellent reversibility and the invariant peak intensity in the initial five cycles, indicating the structural reversibility from O3 to H1‐3 under 4.5–4.6 V. To accurately reflect the structural changes during charging and discharging in situ and ex situ XRD were also conducted. In Figure S13 (Supporting Information), the peak at 18.4° is attributed to the (003) crystal, and a new peak appears at 19.3° when the LCO cathode is charged to 4.6 V, corresponding to the phase transition from O3 to H1‐3. The variation of the 003 peak is associated with the value of c, which responds to the volume change during the charging and discharging of the LCO cathode.^[^ ^36^ ^]^ As shown in Figure 4c,d, the amplitude of the (003) peak change is greater for commercial electrolytes (0.96°) than for eutectogel electrolytes (0.84°). This result illustrates that eutectogel electrolyte has a much smaller degree of volume change, which maintains structural stability and avoids severe structural deterioration. Moreover, the peak at 19.3° remains after 10 and 50 cycles of the eutectogel electrolyte, while it disappears after 10 and 50 cycles of the commercial electrolyte (Figure 4e), confirming that the LCO cathode is unable to undergo the H1‐O3 phase transition after cycling in a commercial electrolyte. During deep lithiation/delithiation, the strength of the TM─O bond is closely related to the structural changes of the LCO. Figure 4f displays the variation of the two peaks at 485 cm^−1^ (O─Co─O, Eg) and 595 cm^−1^ (Co─O, A1g) after ten cycles.^[^ ^9^ ^]^ The peak intensity of the commercial electrolyte is significantly weaker, while the eutectogel electrolyte exhibits excellent structural reversibility after cycling, which should be ascribed to the smaller volume changes of eutectogel electrolyte during cycling, inhibiting the irreversible breaking of O─Co─O and Co─O. Cobalt dissolution is related to the capacity decay of the LCO cathode at high voltages. The dissolved Co contents in the commercial and eutectogel electrolytes were determined after ten cycles between 3.0–4.6 V. The Co content in the eutectogel electrolyte is 5.27 ppm, which is lower than the 28.34 ppm in commercial electrolytes, indicating that the LCO structure maintains stability after cycling (Figure S14, Supporting Information).

The unsatisfactory cycling performances of commercial and eutectic electrolytes can be attributed to not only the continuous growth of lithium dendrites but also the inability to establish a stable electrode/electrolyte interface, thereby causing continuous side reactions. The main effects induced by different electrolytes at the electrodes can be further explained by electrochemical impedance spectroscopy (EIS). As depicted in Figure S15 (Supporting Information), there is a distinct variation in the charge transfer resistance (*R_ct_*) between the different electrolytes. The *R_ct_* of the cell cycled in eutectogel electrolyte is 253 Ω after 500 cycles under high voltage, which is significantly lower than that of eutectic electrolyte (425 Ω) and commercial electrolyte (2255 Ω). This result confirms that the eutectogel electrolyte plays a pivotal role in promoting the formation of a more stable interfacial layer and effectively inhibiting excessive electrolyte decomposition, ultimately improving Li^+^ kinetics across the interface.

**Figure 5:**
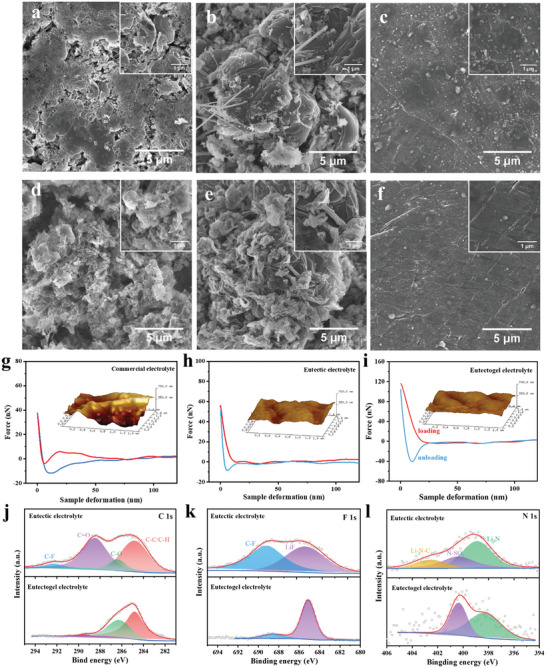
SEM images of Li metal surface after a–c) 50 cycles and d–e) 100 cycles in (a, d) commercial electrolyte, b, e) eutectic electrolyte, and c, f) eutectogel electrolyte. AFM image (inset) and indentation curves of Li metal after 100 cycles in g) commercial electrolyte, h) eutectic electrolyte, and i) eutectogel electrolyte. XPS characterization of lithium metal in eutectic and eutectogel electrolytes: j) C 1s, k) F 1s, and l) N 1s.

To gain a deeper insight into the significantly improved cycling performance achieved with the eutectogel electrolyte, the lithium metal anodes subjected to different cycles were characterized by SEM and atomic force microscopy (AFM) (**Figure** **5**). The lithium metal surface cycled in commercial electrolyte exhibits a loose and porous structure even with Li dendrites after 50 cycles (Figure 5a). Moreover, a large number of mossy lithium and lithium dendrites can be clearly observed after 100 cycles (Figure 5b). The formation of mossy and dendritic Li during plating leads to continuous corrosion of reactive Li and electrolyte depletion. These can cause sudden increased voltages and impedances, resulting in inferior cycling performance.^[^ ^21^ ^]^ The spikes of Li dendrites can pierce the separator and raise safety concerns. In eutectic electrolytes, the Li metal surface is very rough with distinct dendritic or mossy Li even only after 50 cycles (Figure 5d,e). By contrast, the eutectogel electrolyte displays an extraordinarily smooth, and flat surface without any dendritic or mossy Li. This compact and uniform layer of lithium deposition is indicative of the formation of an extremely robust and stable SEI film in the eutectogel electrolyte, which effectively shields the lithium metal anode (Figures 5g,h). TheAFM images in the inset of Figures 5c,f,i further highlight the smoother surface of lithium metal cycled in eutectogel electrolytes compared to that cycled in commercial electrolytes and eutectic electrolytes. In addition, the loading and unloading curves on cycled Li metal in commercial electrolytes are almost overlapping, and the peak force and slope are shown in Table S6 (Supporting Information). The high slope of the curve demonstrates that the SEI layer formed in commercial electrolytes is stiff and brittle. In stark contrast, the lower peak force and slope can be observed for eutectogel electrolytes, indicating the SEI layer is more elastic and less prone to fracture during the plating/stripping process.^[^ ^37^ ^]^

We further elucidate the different components of SEI in eutectic electrolytes and eutectogel electrolytes by XPS characterization. As shown in Figure 5j–l, a clear difference can be observed in the C1s spectra, with the presence of C─F and C═O peaks in a eutectic electrolyte, but not in eutectogel electrolyte. This C─F peak mainly originates from the decomposition of LiTFSI, indicating that the eutectogel electrolyte can effectively inhibit the decomposition of lithium salts.^[^ ^32^ ^]^ In addition, the eutectogel electrolyte has a higher content of LiF (88%) than the eutectic electrolyte (53%), primarily originating from the reduction of FEC and lithium salts. LiF has excellent electronic insulation and mechanical strength, which reduces the reaction between lithium metal and electrolyte and inhibits dendrite growth.^[^ ^38^ ^]^ In the N 1s spectra, the Li_3_N peak is observed in both the eutectic electrolyte and the eutectogel electrolyte, which can promote the rapid migration of Li^+^ in SEI and induce uniform lithium deposition.^[^ ^39^, ^40^ ^]^ It is noteworthy that the additional organic peak Li─N─C is present in the eutectic electrolyte.^[^ ^41^ ^]^ These results indicate that the formation of SEI with inorganic‐rich LiF and Li_3_N in the eutectogel electrolyte effectively reduces electrolyte decomposition and inhibits lithium dendrite growth.

**Figure 6:**
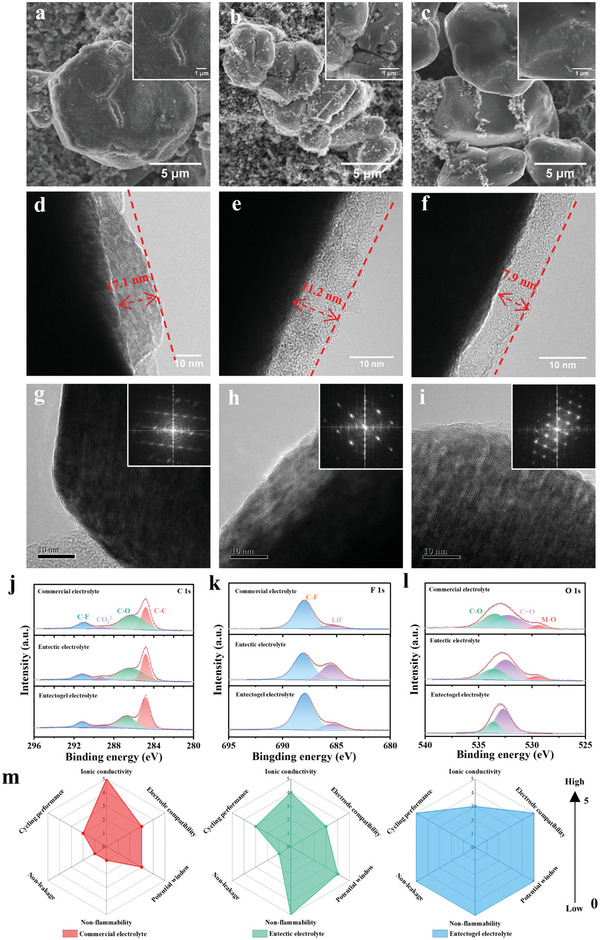
SEM and TEM images of LCO cathode after 100 cycles in (a, d, g) commercial electrolyte, (b, e, h) eutectic electrolyte, and c, f, i) eutectogel electrolyte. XPS characterization of LCO cathode after cycling in different electrolytes: (j) C 1s, (k) F 1s, and (l) O 1s. (m) Comparison of battery performance with three electrolyte systems.

The morphology evolution of the different electrolytes on the LCO cathode is also essential for cycling performance. As depicted in **Figures** **6**a,b and S17 (Supporting Information), the surface of the fresh LCO cathode is smooth and clean. However, noticeable cracks and rough surfaces appear on the LCO cathode after 100 cycles in commercial electrolyte and eutectic electrolyte. This is primarily due to the structural destruction of the LCO cathodes and the accumulation of by‐products of electrolyte decomposition. The thick accumulation layer can lead to increased interfacial resistance, hindering Li^+^ diffusion kinetics and resulting in increased polarization during cell cycling.^[^ ^42^ ^]^ In contrast, the eutectogel electrolyte maintains a smooth surface of the LCO cathode without cracks (Figure 6c). This result indicates eutectogel electrolytes can effectively protect the LCO cathode and significantly inhibit excessive electrolyte decomposition at high voltages. As expected, the CEI film formed in eutectogel electrolyte (7.9 nm) is relatively thinner and more uniform than that of commercial electrolytes (17.1 nm) and eutectic electrolytes (11.2 nm) (Figure 6d–f), which is consistent with its superior cycling performance as discussed above.

The lattice of the LCO cathode was analyzed by TEM after 50 cycles. As shown in Figure 6g–i, the eutectogel electrolyte exhibits a clearer lattice and electron diffraction than the commercial electrolyte and eutectic electrolyte, indicating that the LCO cathode still maintains good structural stability after cycling, whereas the commercial electrolyte and eutectic electrolyte may be deteriorated due to the side‐reaction layer covering the surface of the LCO cathode, which is in agreement with the results of the XRD and Raman analyses.

XPS spectra were further performed to analyze the chemical composition of the surface layer on cycled LCO cathodes (Figure 6j,k). In the F 1s spectra, the signals of LiF (685.3 eV) and C─F (688.2 eV) can be clearly detected.^[^ ^43^ ^]^ The LiF is mainly generated from the oxidation decomposition of LiTFSI or LiDFOB, and notably, its presence is less pronounced in the eutectogel electrolyte compared to the eutectic electrolyte. This result validates the suppression of malignant consumption of lithium salts. In O 1s spectra, the M─O signal (529.6 eV) can be clearly observed in commercial electrolyte and eutectic electrolytes, but disappears in eutectogel electrolytes, indicating that the CEI films formed in commercial and eutectic electrolytes are unable to passivate the active cathode surface effectively.^[^ ^5^ ^]^ As a result, a thin and uniform CEI film can be formed in the eutectogel electrolyte, effectively inhibiting excessive electrolyte decomposition and protecting the LCO cathode. Based on the above results, Figure 6m compares the properties of the three electrolytes in six aspects. Except for the unsatisfactory ionic conductivity, the eutectogel electrolyte displays a comprehensive range of attractive features superior to the commercial electrolyte and eutectic electrolyte, including non‐leakage, nonflammability, wide electrochemical window, high electrode compatibility, and excellent high‐voltage cycling performance.

## 3 Conclusion

In summary, a novel eutectogel electrolyte has been designed and prepared to stabilize the electrode–electrolyte interface for high‐voltage LCO||Li cells. The eutectogel electrolyte enables the outstanding cycling performance of LCO||Li cells with capacity retention of 72.5% and 87.7% after 1500 and 100 cycles at high charging voltages of 4.45 and 4.6 V, respectively. The significantly improved performance of the eutectogel electrolyte is attributed to the stabilization of both the lithium metal anode and the LCO cathode under high voltage. On the one hand, the lithium metal anode forms a stable SEI with inorganic‐rich LiF and Li_3_N, which inhibits the growth of lithium dendrites. On the other hand, excessive electrolyte decomposition is suppressed and a CEI with strong passivation capability is formed, maintaining the structural stability of the LCO cathode at high voltage. This work provides a pathway to design new electrolytes for high voltage and high energy lithium metal batteries with high safety and long cycle life. Benefiting from the unique interfacial optimization effect of eutectogel electrolytes, we predict that this will boost the applications of all high‐voltage cathode material (including high‐nickel and lithium‐rich cathodes).

## Conflict of Interest

The authors declare no conflict of interest.

## Author Contributions

J.Z., J.Z., and L.C. conceived the project. W.W. and D.L. designed the project. W.W. conducted most of the synthetic experiments. D.L. led the electrochemical experiments and analysis. Y.B. and J.Z. conducted the density functional theory computational study. W.W. and D.L. wrote the original draft of the manuscript. C.G. and H.W. reviewed and edited the manuscript. All authors discussed and commented on the manuscript.

## Supporting information

Supporting Information

## Data Availability

The data that support the findings of this study are available from the corresponding author upon reasonable request.
