# Supplementary material for: Eutectogel Electrolyte Constructs Robust Interfaces for High‐Voltage Safe Lithium Metal Battery
Source: Adv Sci (Weinh). 2024 Apr 19;11(23):2310136. doi: 10.1002/advs.202310136 (PMC11187895; doi:10.1002/advs.202310136)
Supplement: Supplementary file 1 — Supporting Information [file ADVS-11-2310136-s001.docx]

Supporting Information **Eutectogel Electrolyte Enables Long Cycling for High-Voltage Safe Lithium Metal Battery**

Wanbao Wu^#^, Deping Li^#^, Chaochao Gao, Hao Wu, Yiyang Bo, Jichuan Zhang,* Lijie Ci,* Jiaheng Zhang*

W. B. Wu

School of Petrochemical Engineering, Changzhou University, 21300, China

Y. Y. Bo, C. C. Gao, H. Wu, J. C. Zhang, Prof. J. H. Zhang

Sauvage Laboratory for Smart Materials, Harbin Institute of Technology (Shenzhen), 518055, China.

Research Centre of Printed Flexible Electronics, School of Materials Science and Engineering, Harbin Institute of Technology (Shenzhen), 518055, China

E-mail: zhangjiaheng@hit.edu.cn

D. P. Li, Prof. L. J. Ci

School of Materials Science and Engineering, Harbin Institute of Technology (Shenzhen), 518055, China.

E-mail: cilijie@hit.edu.cn

**Methods**

**Material:** Lithium bis(trifluoromethanesulfonyl)imide (LiTFSI), lithium difluoro(oxalato)borate (LiDFOB), succinonitrile (SN), fluoroethylene carbonate (FEC) were purchased from Aladdin Reagent Co., Ltd. The commercial electrolyte 1 M LiPF_6_ EC:DEC (1:1 by vol) was purchased from Guangdong Canrd New Energy Technology Co., Ltd. and used as a reference. LCO cathodes were obtained from the Guangdong Canrd New Energy Technology Co., Ltd. All reagents were used without purification.

**Preparation of electrolytes.**

**Preparation of eutectic electrolyte:** eutectic electrolyte was prepared by mixing LiTFSI: LiDFOB: SN in a molar ratio of 0.8:0.2:10 at room temperature until a transparent solution was obtained, and 10 wt.% FEC was added as an additive (denoted as eutectic electrolyte).

**Preparation of eutectogel electrolyte:** A solution with 10 wt.% PEO was prepared by dissolving 1 g of PEO in 9 g of NMP and stirred at 70 °C until completely dissolved. The B solution was obtained by adding 1.0 g P(VDF-HFP) with 0.5 g EMITFSI to 6.0 mL of anhydrous acetone and stirring vigorously until completely dissolved. Then, PEO with a mass ratio of 30 wt.% of PVDF-HFP was added drop by drop to solution B and stirred at 70°C for 0.5 h. After cooling to room temperature, the mixture was casted on an Al foil by a doctor blade to form a uniform membrane, and then dried at 60 °C for 2 h, the membrane is defined as PP-IL. Finally, the membrane was peeled off and cut into disks of 19 mm diameter, then transferred to an Ar-filled glove box and then immersed in eutectic electrolyte for 24 hours (denoted as eutectogel electrolyte).

**Electrochemical measurements**

The LCO cathode materials were fabricated by mixing the active material, super P, and PVDF in a weight ratio of 8:1:1 with NMP as the solvent. The slurry was mixed in a high-speed disperser (FA25 superfine homogenizer) for 30 min and then coated on Al foil with an active material mass loading of 2-2.5 mg cm^−2^. The high loading of LCO cathode was 10 mg cm^−2^. Glass fiber (Whatman, GF/A) and Celgard 2325 were used as the separators for eutectic electrolyte and commercial electrolyte, respectively. The electrodes were punched into 13 mm diameter disks and dried under vacuum at 80 °C for 12 h before use. For cell testing, 80 µL of the eutectic electrolyte and 30 µL of the commercial electrolyte were used. Electrochemical tests of Cu||Li, Li||Li, LCO||Li cells were performed using a Neware battery test system (Neware Technology Co. Ltd. Shenzhen). The current densities of LCO||Li cells were set as 1 C = 178 mA g^−1^. Linear sweep voltammetry (LSV), cyclic voltammetry (CV), and electrochemical impedance spectroscopy (EIS) were performed using a CHI 760D electrochemical workstation.

**Characterization.**

Thermogravimetric analysis (TGA) was carried out at a heating rate of 10 °C min^-1^ over a temperature range from 30 °C to 600 °C under a nitrogen atmosphere. The coordination structure of the eutectic electrolytes and the ionic-dipole interaction between EMITFSI and polymeric matrix were investigated by Raman spectroscopy (Horiba, LabRAM HR800, American) and Fourier transform infrared (FTIR) spectroscopy (IRTracer-100 spectrometer, Japan). The Li deposited morphology was observed by field emission-scanning electron microscopy (FE-SEM) (Hitachi, SU8010, Japan) after Li deposited on Cu substrate. X-ray photoelectron spectroscopy (XPS) was carried out to analyzed the components of the electrode-electrolyte interface on a PHI 5000 VersaProbe II spectrometer with monochromatic Al Kα X-ray radiation. Transmission electron microscopy (TEM) was carried out using a JEM 3200FS instrument at 300kV. All post-test analyses were cleaned three times with DMC solvent after cell disassembly to remove residual lithium salts or impurities from the surface.

**Computational details.**

All-atom molecular dynamics (MD) simulations were executed utilizing the Gromacs 2020 program, employing a general AMBER force field with RESP charges^[1]^. In each case, the system initiated from a relaxed liquid configuration at 298 K. The simulation process commenced with a 10 ns NPT run for equilibrium MD. Subsequently, a 50 ns MD run was conducted in the NVT ensemble for further equilibrium, followed by an additional 50 ns NVT simulation to collect the necessary data. To visualize snapshots, solvation structures, and radial distribution functions, the Visual Molecular Dynamics (VMD) software^[2]^ was employed. Molecular structure optimization was achieved using the ORCA 5.0.1 package^[3]^ at the B3LYP/def2-TZVP(-f) level^[4]^. Each optimized molecule was scrutinized for minimum energy, ensuring an absence of imaginary frequency. Electrostatic potential (ESP)^[5]^ and reduced density gradient (RDG)^[6]^ analyses were carried out using Multiwfn^[7]^ and VMD, respectively.

**
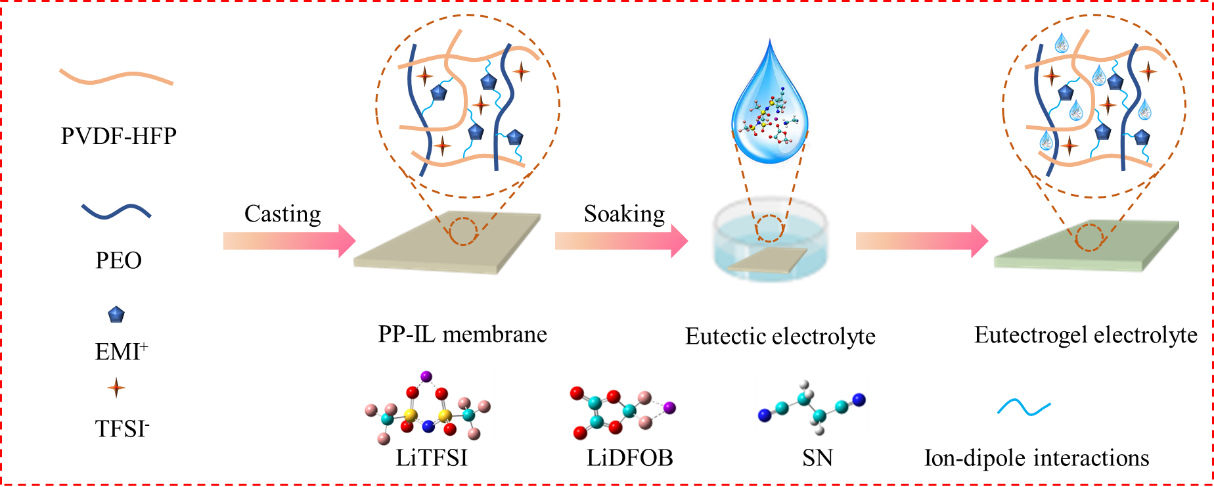
**

Figure S1 Schematic diagram of the synthesis of eutectogel electrolyte

**
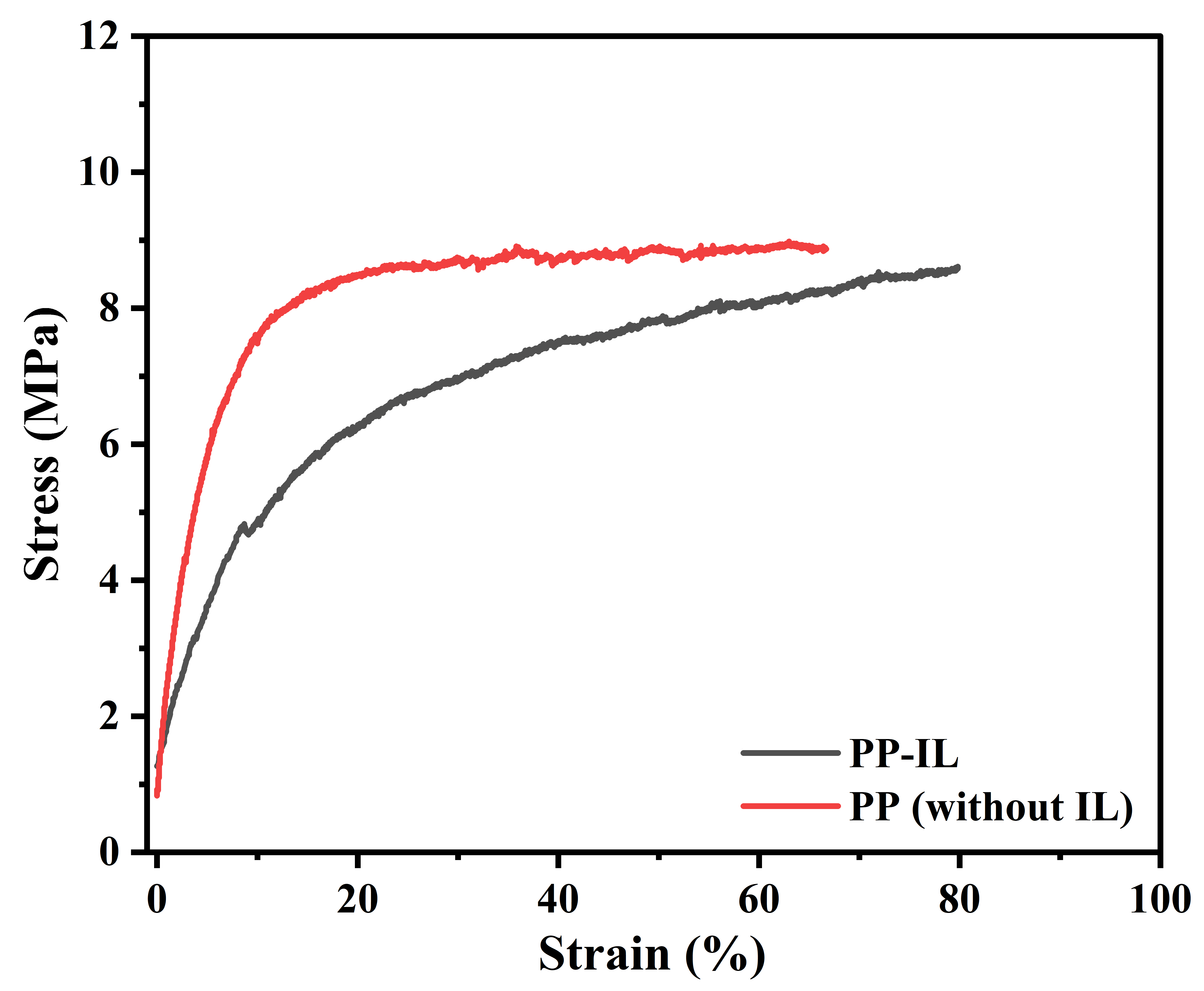
**

Figure S2 The stress-strain curve of PP-IL and PP membrane.

**
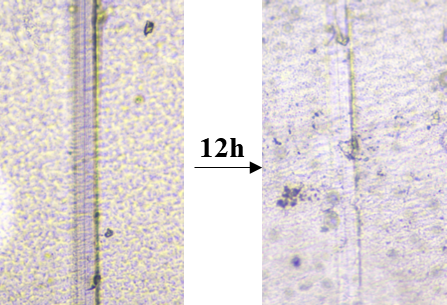
**

Figure S3 Optical microscopy of the self-healing capability of a scratched eutectogel electrolyte menbrane after immersion in a eutectic electrolyte for 12 hours at room temperature.

Figure S4 FTIR spectra of EMITFSI, PVDF-HFP, and PP-IL membranes.

As shown in Figure S4, the peaks 1400 cm^-1^ (-C-F stretching) of PVDF-HFP and 1166 cm^-1^ (imidazole ring stretching) of EMITFSI shifted to 1403 cm^-1^ and 1172 cm^-1^ after introduction of EMITFSI. This indicates that the strong interaction between imidazole cation and PVDF-HFP.

**
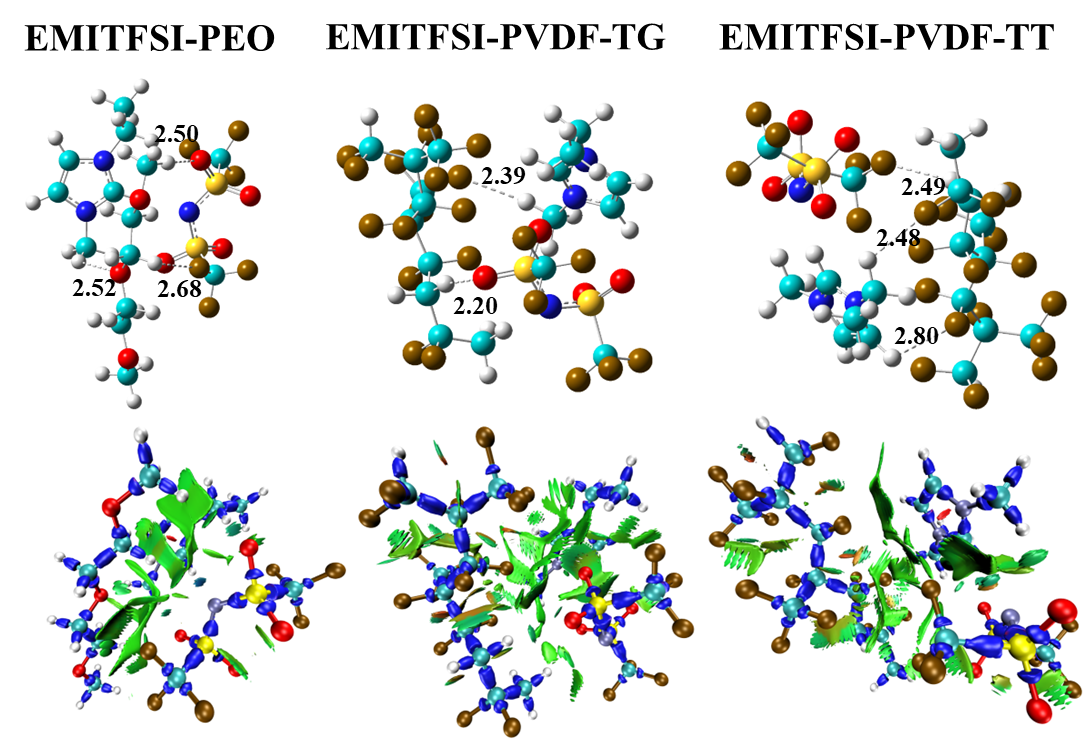
**

Figure S5 DFT geometry optimization of (a) EMITFSI and PEO, (b) EMITFSI and PVDF-HFP (TG) and (c) EMITFSI and PVDF-HFP (TT). Gradient iso-surfaces of (d) EMITFSI and PEO, (e) EMITFSI and PVDF-HFP (TG) and (f) EMITFSI and PVDF-HFP (TT).

The density functional theory (DFT) geometry optimization verifies the short distance between F atom (from PVDF-HFP) and H atom (from EMI^+^) or between H atom (from PVDF-HFP) and O atom (from TFSI-), indicating the existence of interaction between PVDF-HFP and EMITFSI. Similar results can be obtained between PEO and EMITFSI.

Figure S6 Current-time curve of Li/eutectic electrolyte/Li symmetrical cells with a 5 mV DC voltage applied.

Figure S7 CE of Li plating/stripping profiles in different electrolytes at a current density of 0.2 mA cm^−2^ and the capacity of 0.2 mAh cm^−2^.


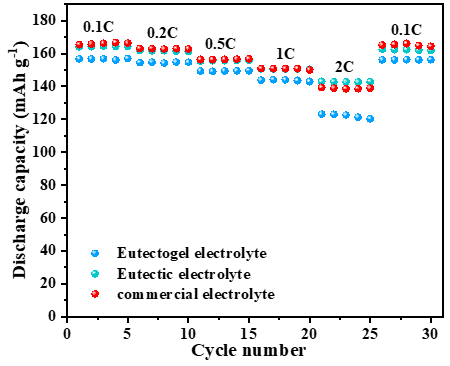


Figure S8 Rate performance of LCO||Li cells with different electrolytes at 3.0–4.45 V.

Figure S9 Voltage spikes observed during cycling of LCO||Li at the 165th cycle with eutectic electrolyte.


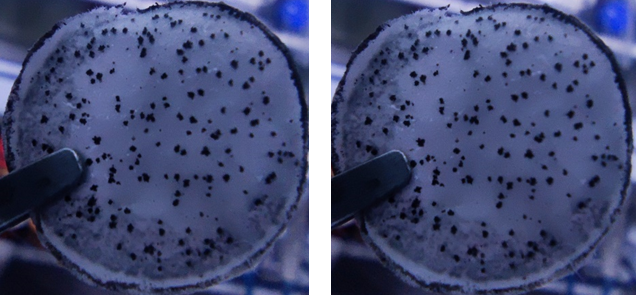


Figure S10 Photograph of both sides of the separator after 165 cycles of the eutectic electrolyte.


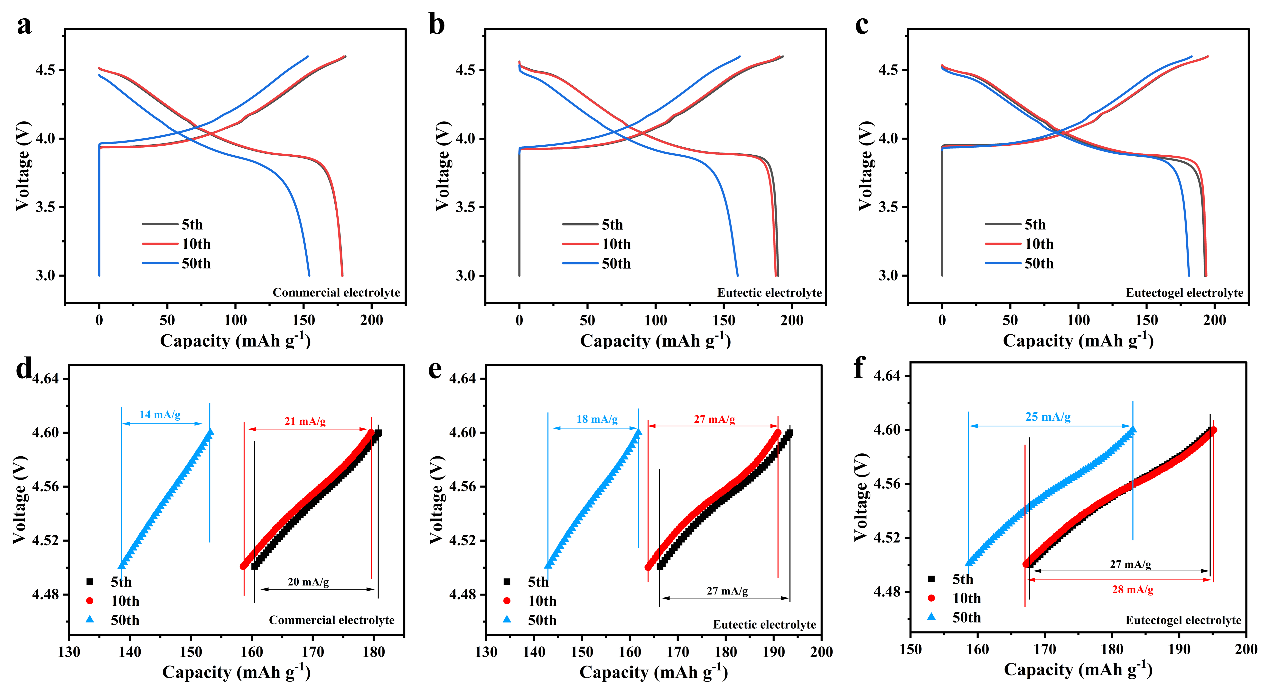


Figure S11 Corresponding charge/discharge curves with different cycles in (a) commercial electrolyte, (b) eutectic electrolyte and (c) eutectogel electrolyte at 3.0-4.6 V. The corresponding charging capacity of different cycles between 4.5 and 4.6V in (d) commercial electrolyte, (e) eutectic electrolyte and (f) eutectogel electrolyte.


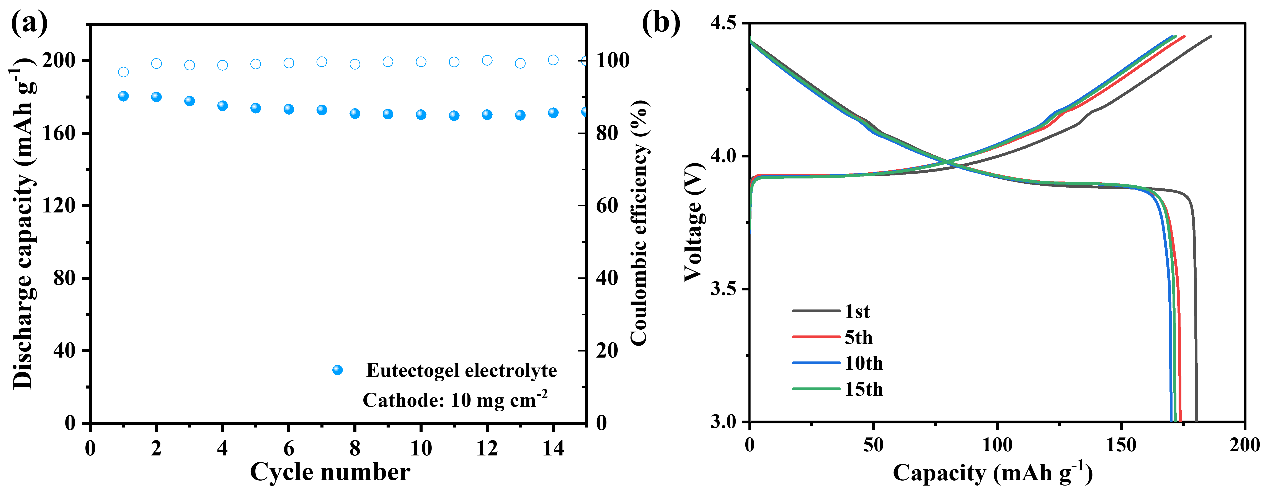


Figure S12 (a) The cycling performance and (b) the charge-discharge profiles of eutectogel electrolyte with high loading under 3.0-4.45V .


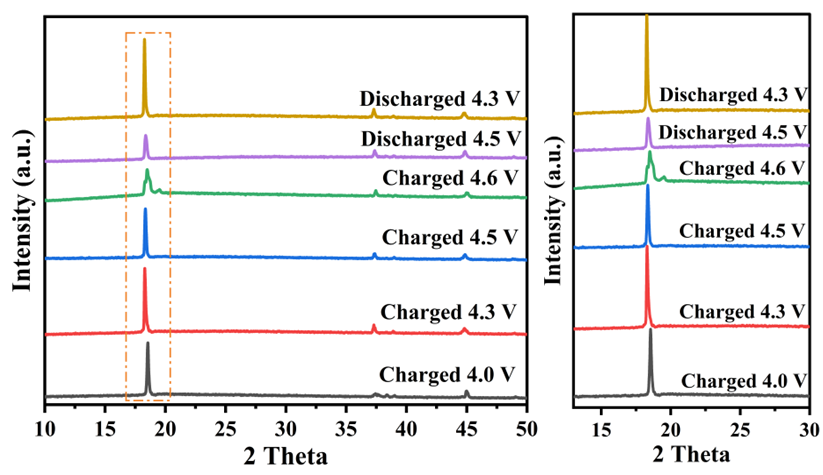


Figure S13 The ex situ XRD patterns of LCO electrolyte during the charging process under 0.1 C.

Figure S14 Contents of transition metal Co ions in different electrolytes after 10 cycles under 3.0-4.6 V.


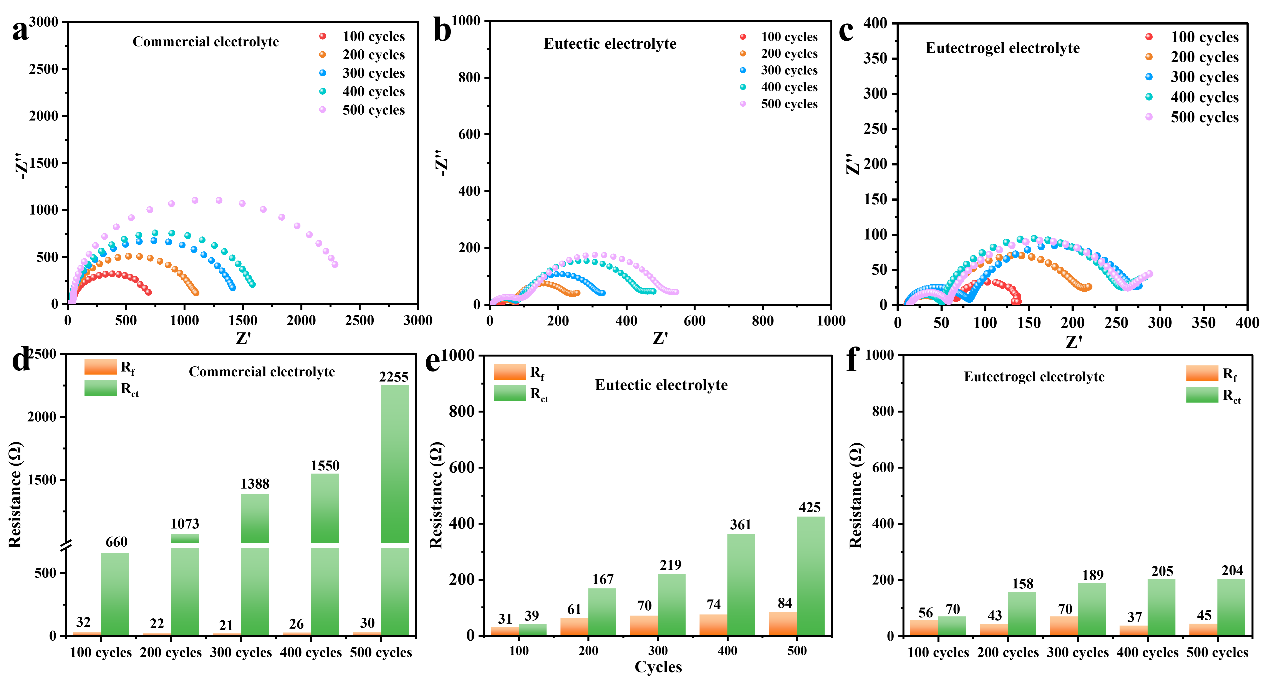


Figure S15 Electrochemical impedance spectra of the cells and the corresponding fitting results of R_f_ and R_ct_ by equivalent circuit after different cycles in (a, d) commercial electrolyte, (b, e) eutectic electrolyte, and (c, f) eutectogel electrolyte.

Figure S16 The content of LiF and C-F in SEI.


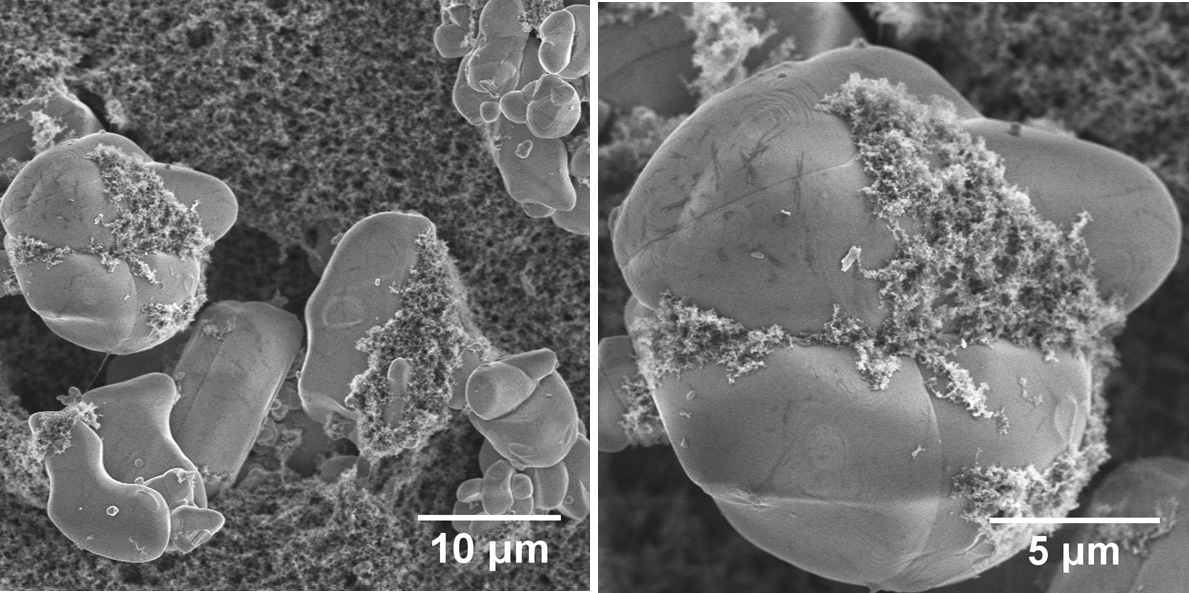


Figure S17 SEM images of fresh LCO electrode.

**Table S1** The adsorbance of eutectic electrolyte in PP-IL membranes

| Sample | 1 | 2 | 3 |
| --- | --- | --- | --- |
| Mass before immersion | 20.3 mg | 24.8 mg | 20.2 mg |
| Mass after immersion | 22.9 mg | 27.4 mg | 22.3 mg |
| Percentage of adsorption | 12.8% | 10.5% | 10.4% |
| Average adsorbance | 11.25% | | |

**Table S2** Comparison of the conductivity of different gel electrolytes

| Electrolyte | Conductivity (mS cm^−1^) | Refer |
| --- | --- | --- |
| HVTPE | 0.15 | ^[8]^ |
| IPL | 0.39 | ^[9]^ |
| PISE | 0.36 | ^[10]^ |
| NPH-GPE | 0.12 | ^[11]^ |
| SIGE | 0.12 | ^[12]^ |
| LiTFSI-IL-P(VDF-HFP) | 0.88 | ^[13]^ |
| CPG | 0.67 | ^[14]^ |
| SMS/[Li(G4)] [TFSA] | 0.17 | ^[15]^ |
| poly(MPC-co-SBVI) | 0.60 | ^[16]^ |
| IGPE | 0.21 | ^[17]^ |
| Eutectic electrolyte | 3.62 | This work |
| Eutectogel electrolyte | 0.63 | This work |

**Table S3** Molecular Dynamics (MD) simulation details for eutectic electrolyte.

| **Electrolyte** | **Eutectic electrolyte** |
| --- | --- |
| No. LiTFSI in box | 80 |
| No. LiDFOB in box | 20 |
| No. SN in box | 1000 |

**Table S4** Comparison of the Coulombic efficiency of different electrolytes

| Electrolyte | Coulombic efficiency | Refer |
| --- | --- | --- |
| LiFSI-DMC-TTE | 98.7% | ^[18]^ |
| LiTFA-FEC/DME | 98.8% | ^[19]^ |
| EM-5Li IL electrolytes | 99.0% | ^[20]^ |
| SGPE | 98.2% | ^[21]^ |
| PHL-LiNO_3_ | 95.9% | ^[22]^ |
| PDOL | 94.2% | ^[23]^ |
| CgTSPE | 90.0% | ^[24]^ |
| mixed-DIPE | 90.0% | ^[25]^ |
| Eutectic electrolyte | 94.9% | This work |
| Eutectogel electrolyte | 94.7% | This work |

**Table S5** Comparison of the electrochemical performance of LCO||Li cells based on different electrolytes

| Electrolyte | Voltage | Rate | Cycle number | Retention | Cathode loading  (mg cm^-2^) | Refer |
| --- | --- | --- | --- | --- | --- | --- |
| LiTFSI-P_13_FSI-TTE | 3.0-4.3 | 0.5 C | 200 | 85% | 5.3 | ^[26]^ |
| DSM-SPE | 3.0-4.3 | 0.1 C (60 ℃) | 100 | 83% | 5.0 | ^[27]^ |
| D-DEE-SPE | 3.0-4.3 | 0.3 | 200 | 88% | 1.3 | ^[28]^ |
| D-DEE-TXE | 3.0-4.45 | 0.3 | 100 | 81% | 2.6 | ^[29]^ |
| 2%LBTB | 3.0-4.4 | 1 C | 300 | 73% | 1.8–2.2 | ^[30]^ |
| SN-DLi-FEC | 3.0-4.4 | 0.5 C | 200 | 85% | 2.0 | ^[31]^ |
| PVDF/PVAC-Based CPE | 3.0-4.5 | 0.5 C | 200 | 85% | 1.5 | ^[32]^ |
| 0.2% ATCN | 3.0-4.5 | 1 C | 200 | 91% | 2.3–2.6 | ^[33]^ |
| D-DES | 3.0-4.5 | 1 C | 750 | 77% | --- | ^[34]^ |
| 2% GTCE additive | 2.75-4.5 | 1 C | 150 | 79% | 2.0 | ^[35]^ |
| Sulfonamide-based electrolyte | 3.0-4.6 | 0.3 C | 100 | 85% | 11.0 | ^[36]^ |
| FEC-HTCN additive | 3.0-4.6 | 1 C | 300 | 75% | 6.0 | ^[37]^ |
| 0.1% KSeCN additive | 3.0-4.6 | 1 C | 750 | 69% | 3.0 | ^[38]^ |
| 1 M LiPF_6_ EC-DEC | 3.0-4.45 | 0.5 C | 900 | 45% | 2.0-2.5 | This work |
| 1 M LiPF_6_ EC-DEC | 3.0-4.6 | 0.5 C | 100 | 67% |  | This work |
| Eutectic electrolyte | 3.0-4.45 | 0.5 C | 1000 | 23% |  | This work |
| Eutectic electrolyte | 3.0-4.6 | 0.5 C | 100 | 68% |  | This work |
| Eutectogel electrolyte | 3.0-4.45 | 0.5 C | 1500 | 73% |  | This work |
| Eutectogel electrolyte | 3.0-4.6 | 0.5 C | 100 | 88% |  | This work |

**Table S6** The results of the AFM analysis.

| **Different electrolyte** | **Slope** | **Peak Force (nN)** |
| --- | --- | --- |
| Commercial electrolyte | -9.53 | -11.59 |
| Eutectic electrolyte | -8.28 | -8.29 |
| Eutectogel electrolyte | -7.65 | -40.20 |

**Reference**

[1] C. I. Bayly, P. Cieplak, W. Cornell, P. A. J. T. J. o. P. C. Kollman, A well-behaved electrostatic potential based method using charge restraints for deriving atomic charges: the RESP model, **1993**, 97, 10269.

[2] W. Humphrey, A. Dalke, K. J. J. o. m. g. Schulten, VMD: visual molecular dynamics, **1996**, 14, 33.

[3] F. J. W. I. R. C. M. S. Neese, The ORCA program system, **2012**, 2, 73.

[4] F. Weigend, R. J. P. C. C. P. Ahlrichs, Balanced basis sets of split valence, triple zeta valence and quadruple zeta valence quality for H to Rn: Design and assessment of accuracy, **2005**, 7, 3297.

[5] T. Lu, F. J. J. o. M. G. Chen, Modelling, Quantitative analysis of molecular surface based on improved Marching Tetrahedra algorithm, **2012**, 38, 314.

[6] E. R. Johnson, S. Keinan, P. Mori-Sánchez, J. Contreras-García, A. J. Cohen, W. J. J. o. t. A. C. S. Yang, Revealing noncovalent interactions, **2010**, 132, 6498.

[7] T. Lu, F. J. J. o. c. c. Chen, Multiwfn: A multifunctional wavefunction analyzer, **2012**, 33, 580.

[8] J. Liu, X. Shen, J. Zhou, M. Wang, C. Niu, T. Qian, C. J. A. a. m. Yan, interfaces, Nonflammable and high-voltage-tolerated polymer electrolyte achieving high stability and safety in 4.9 V-class lithium metal battery, **2019**, 11, 45048.

[9] D. Cai, X. Wu, J. Xiang, M. Li, H. Su, X. Qi, X. Wang, X. Xia, C. Gu, J. J. C. E. J. Tu, Ionic-liquid-containing polymer interlayer modified PEO-based electrolyte for stable high-voltage solid-state lithium metal battery, **2021**, 424, 130522.

[10] H. Li, Y. Du, X. Wu, J. Xie, F. J. A. F. M. Lian, Developing “polymer‐in‐salt” high voltage electrolyte based on composite lithium salts for solid‐state li metal batteries, **2021**, 31, 2103049.

[11] J. Jie, Y. Liu, L. Cong, B. Zhang, W. Lu, X. Zhang, J. Liu, H. Xie, L. J. J. o. e. c. Sun, High-performance PVDF-HFP based gel polymer electrolyte with a safe solvent in Li metal polymer battery, **2020**, 49, 80.

[12] L. Yu, S. Guo, Y. Lu, Y. Li, X. Lan, D. Wu, R. Li, S. Wu, X. J. A. E. M. Hu, Highly tough, Li‐metal compatible organic–inorganic double‐network solvate ionogel, **2019**, 9, 1900257.

[13] T. Chen, W. Kong, Z. Zhang, L. Wang, Y. Hu, G. Zhu, R. Chen, L. Ma, W. Yan, Y. J. N. E. Wang, Ionic liquid-immobilized polymer gel electrolyte with self-healing capability, high ionic conductivity and heat resistance for dendrite-free lithium metal batteries, **2018**, 54, 17.

[14] J.-H. Baik, S. Kim, D. G. Hong, J.-C. J. A. a. m. Lee, interfaces, Gel Polymer electrolytes based on polymerizable lithium salt and poly (ethylene glycol) for lithium battery applications, **2019**, 11, 29718.

[15] Y. Kitazawa, K. Iwata, R. Kido, S. Imaizumi, S. Tsuzuki, W. Shinoda, K. Ueno, T. Mandai, H. Kokubo, K. J. C. o. M. Dokko, Polymer electrolytes containing solvate ionic liquids: A new approach to achieve high ionic conductivity, thermal stability, and a wide potential window, **2018**, 30, 252.

[16] A. J. D’Angelo, M. J. J. C. o. M. Panzer, Design of stretchable and self-healing gel electrolytes via fully zwitterionic polymer networks in solvate ionic liquids for Li-based batteries, **2019**, 31, 2913.

[17] L. Chen, J. Fu, Q. Lu, L. Shi, M. Li, L. Dong, Y. Xu, R. J. C. E. J. Jia, Cross-linked polymeric ionic liquids ion gel electrolytes by in situ radical polymerization, **2019**, 378, 122245.

[18] N. Piao, X. Ji, H. Xu, X. Fan, L. Chen, S. Liu, M. N. Garaga, S. G. Greenbaum, L. Wang, C. J. A. E. M. Wang, Countersolvent electrolytes for lithium‐metal batteries, **2020**, 10, 1903568.

[19] Z. Wang, F. Qi, L. Yin, Y. Shi, C. Sun, B. An, H. M. Cheng, F. J. A. e. m. Li, An anion‐tuned solid electrolyte interphase with fast ion transfer kinetics for stable lithium anodes, **2020**, 10, 1903843.

[20] H. Sun, G. Zhu, Y. Zhu, M. C. Lin, H. Chen, Y. Y. Li, W. H. Hung, B. Zhou, X. Wang, Y. J. A. M. Bai, High‐safety and high‐energy‐density lithium metal batteries in a novel ionic‐liquid electrolyte, **2020**, 32, 2001741.

[21] Y.-H. Lin, R. Subramani, Y.-T. Huang, Y.-L. Lee, J.-S. Jan, C.-C. Chiu, S.-S. Hou, H. J. J. o. M. C. A. Teng, Highly stable interface formation in onsite coagulation dual-salt gel electrolyte for lithium-metal batteries, **2021**, 9, 5675.

[22] H. Duan, Y. You, G. Wang, X. Ou, J. Wen, Q. Huang, P. Lyu, Y. Liang, Q. Li, J. J. N.-M. L. Huang, Lithium-Ion Charged Polymer Channels Flattening Lithium Metal Anode, **2024**, 16, 78.

[23] Y. Zhang, J. Huang, G. Wang, Y. Dou, D. Yuan, L. Lin, K. Wu, H. K. Liu, S.-X. Dou, C. J. N. Wu, A quasi-solid polymer electrolyte initiated by two-dimensional functional nanosheets for stable lithium metal batteries, **2023**, 15, 9700.

[24] M. Zhang, A. L. Gui, W. Sun, J. Becking, O. Riedel, X. He, D. Berghus, V. Siozios, D. Zhou, T. J. J. o. T. E. S. Placke, High capacity utilization of Li metal anodes by application of celgard separator-reinforced ternary polymer electrolyte, **2019**, 166, A2142.

[25] W.-J. Chen, C.-X. Zhao, B.-Q. Li, Q. Jin, X.-Q. Zhang, T.-Q. Yuan, X. Zhang, Z. Jin, S. Kaskel, Q. J. E. Zhang, E. Materials, A mixed ether electrolyte for lithium metal anode protection in working lithium–sulfur batteries, **2020**, 3, 160.

[26] S. Lee, K. Park, B. Koo, C. Park, M. Jang, H. Lee, H. J. A. F. M. Lee, Safe, stable cycling of lithium metal batteries with low‐viscosity, fire‐retardant locally concentrated ionic liquid electrolytes, **2020**, 30, 2003132.

[27] C. Wang, T. Wang, L. Wang, Z. Hu, Z. Cui, J. Li, S. Dong, X. Zhou, G. Cui, Differentiated Lithium Salt Design for Multilayered PEO Electrolyte Enables a High-Voltage Solid-State Lithium Metal Battery, Adv Sci (Weinh), **2019**, 6, 1901036.

[28] H. Wu, B. Tang, X. Du, J. Zhang, X. Yu, Y. Wang, J. Ma, Q. Zhou, J. Zhao, S. J. A. S. Dong, LiDFOB initiated in situ polymerization of novel eutectic solution enables room‐temperature solid lithium metal batteries, **2020**, 7, 2003370.

[29] J. Zhang, H. Wu, X. Du, H. Zhang, L. Huang, F. Sun, T. Liu, S. Tian, L. Zhou, S. J. A. E. M. Hu, Smart deep eutectic electrolyte enabling thermally induced shutdown toward high‐safety lithium metal batteries, **2023**, 13, 2202529.

[30] Z. Sun, H. Zhou, X. Luo, Y. Che, W. Li, M. Xu, Design of a novel electrolyte additive for high voltage LiCoO2 cathode lithium-ion batteries: Lithium 4-benzonitrile trimethyl borate, Journal of Power Sources, **2021**, 503.

[31] C. Fu, Y. Ma, S. Lou, C. Cui, L. Xiang, W. Zhao, P. Zuo, J. Wang, Y. Gao, G. Yin, A dual-salt coupled fluoroethylene carbonate succinonitrile-based electrolyte enables Li-metal batteries, Journal of Materials Chemistry A, **2020**, 8, 2066.

[32] X. Yu, L. Wang, J. Ma, X. Sun, X. Zhou, G. Cui, Selectively Wetted Rigid–Flexible Coupling Polymer Electrolyte Enabling Superior Stability and Compatibility of High‐Voltage Lithium Metal Batteries, Advanced Energy Materials, **2020**, 10.

[33] D. Ruan, M. Chen, X. Wen, S. Li, X. Zhou, Y. Che, J. Chen, W. Xiang, S. Li, H. Wang, X. Liu, W. Li, In situ constructing a stable interface film on high-voltage LiCoO2 cathode via a novel electrolyte additive, Nano Energy, **2021**, 90.

[34] Z. Hu, F. Xian, Z. Guo, C. Lu, X. Du, X. Cheng, S. Zhang, S. Dong, G. Cui, L. Chen, Nonflammable Nitrile Deep Eutectic Electrolyte Enables High-Voltage Lithium Metal Batteries, Chemistry of Materials, **2020**, 32, 3405.

[35] Z. Zhang, Z. Huang, F. Liu, Y. Song, Q. Mao, X. Fan, M. Bai, B. Hong, Y. J. C. Lai, Glycerol Tris (2‐cyanoethyl) Ether as an Electrolyte Additive to Enhance the Cycling Stability of Lithium Cobalt Oxide Cathode at 4.5 V, **2021**, 8, 4589.

[36] W. Xue, R. Gao, Z. Shi, X. Xiao, W. Zhang, Y. Zhang, Y. G. Zhu, I. Waluyo, Y. Li, M. R. Hill, Z. Zhu, S. Li, O. Kuznetsov, Y. Zhang, W.-K. Lee, A. Hunt, A. Harutyunyan, Y. Shao-Horn, J. A. Johnson, J. Li, Stabilizing electrode–electrolyte interfaces to realize high-voltage Li||LiCoO2 batteries by a sulfonamide-based electrolyte, Energy & Environmental Science, **2021**, 14, 6030.

[37] X. Yang, M. Lin, G. Zheng, J. Wu, X. Wang, F. Ren, W. Zhang, Y. Liao, W. Zhao, Z. J. A. F. M. Zhang, Enabling Stable High‐Voltage LiCoO2 Operation by Using Synergetic Interfacial Modification Strategy, **2020**, 30, 2004664.

[38] A. Fu, J. Lin, Z. Zhang, C. Xu, Y. Zou, C. Liu, P. Yan, D.-Y. Wu, Y. Yang, J. J. A. E. L. Zheng, Synergistical stabilization of Li metal anodes and LiCoO2 cathodes in high-voltage Li∥ LiCoO2 batteries by potassium selenocyanate (KSeCN) additive, **2022**, 7, 1364.
